# Thromboelastometry versus standard coagulation tests versus restrictive protocol to guide blood transfusion prior to central venous catheterization in cirrhosis: study protocol for a randomized controlled trial

**DOI:** 10.1186/s13063-017-1835-5

**Published:** 2017-02-27

**Authors:** Leonardo Lima Rocha, Camila Menezes Souza Pessoa, Ary Serpa Neto, Rogerio Ruscitto do Prado, Eliezer Silva, Marcio Dias de Almeida, Thiago Domingos Correa, on behalf of the POCKET Trial Investigators

**Affiliations:** 1 Adult Critical Care Unit, Hospital Israelita Albert Einstein, São Paulo, Brazil; 2 Liver Transplant Program, Hospital Israelita Albert Einstein, São Paulo, Brazil

**Keywords:** Blood coagulation tests, Blood transfusion, Catheterization, Central venous, Clinical trial, Critical care, Hemostatic disorders, Liver failure, Thromboelastography, Ultrasonography, Vascular access devices

## Abstract

**Background:**

Liver failure patients have traditionally been empirically transfused prior to invasive procedures. Blood transfusion is associated with immunologic and nonimmunologic reactions, increased risk of adverse outcomes and high costs. Scientific evidence supporting empirical transfusion is lacking, and the best approach for blood transfusion prior to invasive procedures in cirrhotic patients has not been established so far.

The aim of this study is to compare three transfusion strategies (routine coagulation test-guided – ordinary or restrictive, or thromboelastometry-guided) prior to central venous catheterization in critically ill patients with cirrhosis.

**Methods/design:**

Design and setting: a double-blinded, parallel-group, single-center, randomized controlled clinical trial in a tertiary private hospital in São Paulo, Brazil. Inclusion criteria: adults (aged 18 years or older) admitted to the intensive care unit with cirrhosis and an indication for central venous line insertion. Patients will be randomly assigned to three groups for blood transfusion strategy prior to central venous catheterization: standard coagulation tests-based, thromboelastometry-based, or restrictive. The primary efficacy endpoint will be the proportion of patients transfused with any blood product prior to central venous catheterization. The primary safety endpoint will be the incidence of major bleeding. Secondary endpoints will be the proportion of transfusion of fresh frozen plasma, platelets and cryoprecipitate; infused volume of blood products; hemoglobin and hematocrit before and after the procedure; intensive care unit and hospital length of stay; 28-day and hospital mortality; incidence of minor bleeding; transfusion-related adverse reactions; and cost analysis.

**Discussion:**

This study will evaluate three strategies to guide blood transfusion prior to central venous line placement in severely ill patients with cirrhosis. We hypothesized that thromboelastometry-based and/or restrictive protocols are safe and would significantly reduce transfusion of blood products in this population, leading to a reduction in costs and transfusion-related adverse reactions. In this manner, this trial will add evidence in favor of reducing empirical transfusion in severely ill patients with coagulopathy.

**Trial registration:**

ClinicalTrials.gov, identifier: NCT02311985. Retrospectively registered on 3 December 2014.

**Electronic supplementary material:**

The online version of this article (doi:10.1186/s13063-017-1835-5) contains supplementary material, which is available to authorized users.

## Background

Central venous catheterization (CVC) is a routine procedure in the intensive care unit (ICU) [1]. In the USA alone, more than 15 million catheter-days are reported annually [2]. Bleeding is the most common complication of CVC [3], with an incidence of between 12 per 1000 [4] and 94 per 1000 catheter insertions [5], and may be life-threatening in severe cases. More recently, the use of ultrasound guidance for CVC has significantly decreased overall mechanical complication rates [6–10] and is safe in patients with coagulopathy [11].

Patients with liver failure are classically believed to be prone to bleeding disorders [12]. Currently, approximately 9 out of 10 ICU physicians empirically transfuse liver failure patients before invasive procedures [13]. However, scientific evidence for this practice is lacking. Recent medical literature evidence now shows that patients with liver failure exhibit a rebalancing ability between anticoagulant and procoagulant factors [12] and have thrombin generation comparable to healthy individuals [14]. Thus, bleeding in those patients may be mainly related to circulatory and endothelial alterations instead of coagulation disorder per se. In spite of that, several physicians rely on standard coagulation tests (SCTs), i.e., prothrombin time (PT), international normalized ratio (INR), activated partial thromboplastin time (aPTT) and platelet count, to guide blood transfusion before CVC in patients with cirrhosis [15, 16].

It has been shown that SCTs reflect a limited view of coagulation status (isolated plasmatic component evaluation) [17, 18] and do not accurately predict bleeding risk in patients with suspected coagulopathy [18–21]. Specifically, patients with liver disease and prolonged PT/aPTT may have coagulation factor levels above the sufficient for adequate clot formation [22]. Moreover, attempts to correct (or normalize) standard coagulation tests by transfusion do not guarantee coagulopathy reversal [23, 24] and to insist on this practice may increase the risk of transfusion-related complications, including death [25].

Rotational thromboelastometry (ROTEM®) is a point-of-care test that evaluates coagulation according to the viscoelastic properties of a whole blood sample [26]. ROTEM® has the advantage of a more comprehensive (clot initiation, formation and stabilization) and faster (online) bedside coagulation evaluation when compared to SCTs [27]. Clinical protocols based on thromboelastometry have been effective in reducing blood transfusion, costs and mortality in diverse clinical settings [28–31] including liver transplantation [32, 33].

General clinical practice guidelines recommend prophylactic transfusion of blood products prior to invasive procedures based on SCTs alterations [34–36]. Nevertheless, there is no consensus as to whether and how blood transfusion should be used before invasive procedures, particularly in patients with cirrhosis. The majority of available data have originated from small clinical trials and observational studies, which limit an evidence-based approach. The culture of blood transfusion is moving towards a rational evidence-based use of blood and its components [37].

This study will compare three blood transfusion protocols (coagulogram-based, thromboelastometry-based and restrictive) prior to CVC in critically ill patients with cirrhosis. We hypothesized that restrictive and/or thromboelastometry-guided transfusion protocols are safe and would decrease the need of blood products transfusion compared to an ordinary coagulogram-based protocol in critically ill patients with cirrhosis submitted to a CVC.

## Methods/design

### Study design

This is a double-blinded, parallel-group, superiority, single-center, randomized controlled clinical trial.

### Study setting

The participating hospital is a private tertiary 657-bed hospital in São Paulo, Brazil, with an active liver transplant program (mean ± standard deviation (SD) 118 ± 15 liver transplants per year). Patients will be screened and recruited in two mixed medical-surgical intensive care units, with 48 beds in total, and 3673 admissions per year.

### Participants

All adult patients (aged 18 years or older) admitted to the ICU with chronic liver failure and an indication for bedside central venous line placement for intravenous (I.V.) medication administration (including vasopressors), hemodialysis and/or hemodynamic monitoring will be eligible for inclusion. Chronic liver failure will be defined as liver cirrhosis of any etiology diagnosed via biopsy or clinical evaluation. The exclusion criteria will be: acute liver failure, an indication for a peripherally inserted central venous catheter, current use of orally administered (e.g., inhibitors of factor Xa, thrombin and vitamin K-dependent factors) or parenterally administered anticoagulants (e.g., nonfractionated heparin or low-molecular-weight heparin) in full therapeutic dosage, von Willebrand disease and enrollment in another clinical trial with blood transfusion as intervention.

### Laboratory procedures

Before a CVC, every included patient will be submitted to blood sampling for SCTs (PT, INR, aPTT and platelet count), ROTEM® analysis, serum fibrinogen, hemoglobin, hematocrit, ionized calcium and arterial pH. Blood samples of 5 mL will be obtained directly from a peripheral vein by applying a light tourniquet to avoid blood stasis, or from a central venous line after discarding the first 5 mL of blood (in the case of an indication for a second CVC, e.g., hemodialysis catheter), or from an arterial line. Blood samples for SCTs, ROTEM® and fibrinogen will be conditioned in citrate tubes (3.2%; Sarstedt Monovette®, Wedel, Germany) and processed immediately. Blood samples for hemoglobin level, hematocrit and platelet count will be conditioned in 2.6-mL tubes containing EDTA KE (Sarstedt Monovette®, Wedel, Germany).

Thromboelastometry analysis (ROTEM® delta, Pentapharm Co., Munich, Germany) will be performed by pipetting 300 μL citrated whole blood and 20 μL 0.2-M calcium chloride (CaCl_2_) with specific activators into a plastic cup [38]. Extrinsically activated (EXTEM), intrinsically activated (INTEM), and fibrinogen polymerization (FIBTEM) assays will be performed. ROTEM® analysis will run for up to 60 min. The parameters coagulation time (CT; s), clot formation time (CFT; s), maximum clot firmness (MCF; mm), alpha angle (α; degrees) and amplitude at 10 min (A10; mm) will be recorded [38].

The EXTEM assay contains a preparation of human recombinant tissue factor, which activates the coagulation cascade and can assess coagulation status via CT_EXTEM_, CFT_EXTEM_ and MCF_EXTEM_ [27]. The FIBTEM assay uses cytochalasin D which is a potent platelet inhibitor and serves to evaluate fibrinogen function and fibrin polymerization [27]. The A10 is a surrogate marker of MCF and can predict coagulopathy with high sensitivity and specificity (1.0 and 0.7, respectively) with the advantage of a faster evaluation of coagulation disorders [39].

Prothrombin time, INR and aPTT (ACL TOP 700 system, Instrumentation Laboratory, Bedford, MA, USA), serum fibrinogen (Clauss method) and platelet count (Sysmex® XN-Series, Sysmex Corporation, Lincolnshire, IL, USA) will be performed according to the hospital’s standard analytical methods.

### Central venous catheterization

All CVCs will be performed with real-time ultrasound (Bard Site ~ Rite® 5 Ultrasound System, Bard Access Systems, Salt Lake City, UT, USA) guidance, using the modified Seldinger (over the guidewire) technique. Only ICU physicians and medical residents trained in ultrasound-guided vascular access will be allowed to perform the procedures. To obtain a valid training status, the ICU physician must have performed at least 50 ultrasound-guided CVCs and at least 12 ultrasound-guided insertions the previous year. The choice of insertion site (jugular, femoral or subclavian) will be at the inserter’s discretion. All procedures will be performed according to best practices guidelines [40] and audited by a staff nurse. A chest X-ray will be ordered after each procedure (except for femoral vein site) to assess catheter positioning and complications. A blinded-to-intervention nurse will inspect the insertion site for local complications within the first 24 h after catheter placement using the HEME tool checklist [41].

Inclusion in the trial does not preclude concomitant care. The researchers will monitor whether patients will receive any blood transfusion related to the procedure within the first 24 h.

### Interventions

Patients will be randomly assigned to three groups, each including a protocol to guide blood product transfusion (fresh frozen plasma (FFP), platelets and/or cryoprecipitate) prior to CVC, namely: (1) a transfusion protocol based on standard coagulation tests – Coagulogram-based group, (2) a transfusion protocol based on rotational thromboelastometry – Thromboelastometry-based group or (3) a restrictive transfusion protocol – Restrictive group – also based on SCTs, but with wider transfusion trigger values.

### Coagulogram-based group

**Fig. 1:**
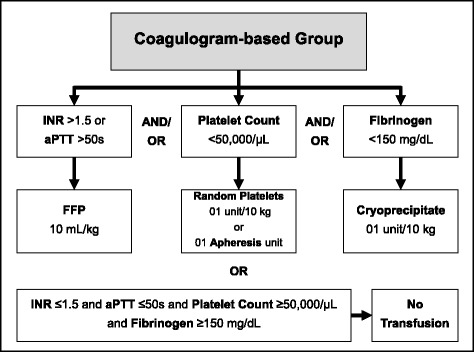
Coagulogram-based blood transfusion protocol. *INR* international normalized ratio; *aPTT* activated thromboplastin time; *FFP* fresh frozen plasma

The Coagulogram-based group (control group) is based on our institutional protocol and takes into account the SCTs PT/INR, aPTT, platelet count and serum fibrinogen levels to guide transfusion prior to CVC (Fig. 1).

Accordingly, if INR ≤1.5, aPTT ≤50 s, platelet count ≥50,000/μL and serum fibrinogen ≥150 mg/dL, no transfusion is indicated. Otherwise, if INR >1.5 or aPTT >50 s, FFP is transfused at 10 mL per kg of body weight; and/or if platelet count <50,000/μL, 1 unit per 10 kg of body weight of random platelets (up to 10 units) or 1 unit of apheresis platelets is transfused; and/or if serum fibrinogen <150 mg/dL, 1 unit per 10 kg of body weight of cryoprecipitate is transfused (up to 10 units).

### Thromboelastometry-based group

**Fig. 2:**
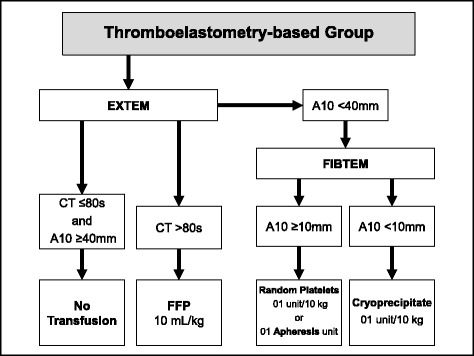
Thromboelastometry-based blood transfusion protocol. *CT* clotting time; *MCF* maximum clot firmness; *A10* amplitude at 10 min; *FFP* fresh frozen plasma

The thromboelastometry-based transfusion protocol uses EXTEM and FIBTEM assays from the ROTEM® and was adapted from Görlinger et al. [42] (Fig. 2). No transfusion is necessary when CT_EXTEM_ is ≤80 s and A10_EXTEM_ is ≥40 mm. The CT_EXTEM_ will be used to assess coagulation factor deficiency. For patients in whom CT_EXTEM_ is >80 s, transfusion of 10 mL per kg of body weight of FFP is performed. If the patient presents an A10_EXTEM_ <40 mm, we further evaluate the A10_FIBTEM_. If A10_FIBTEM_ is ≥10 mm (indicating adequate fibrinogen function), random platelet units (1 unit per 10 kg of body weight; maximum 10 units) or 1 unit of apheresis platelets is transfused. Otherwise, if A10_FIBTEM_ is <10 mm (indicating fibrinogen deficiency), cryoprecipitate (1 unit per 10 kg of body weight; maximum 10 units) is transfused.

### Restrictive group

**Fig. 3:**
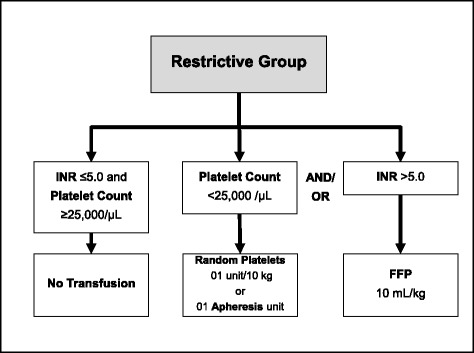
Restrictive blood transfusion protocol. *INR* international normalized ratio; *FFP* fresh frozen plasma

The restrictive transfusion protocol is also based on SCTs, but it uses wider transfusion triggers and it does not take into account serum fibrinogen and aPTT levels (Fig. 3). If INR is ≤5.0 and platelet count is ≥25,000/μL, no transfusion is required. If INR is >5.0, FFP is transfused at 10 mL per kg of body weight; and/or platelet count is <25,000/μL, random platelet units (1 unit per 10 kg of body weight; maximum 10 units) or 1 unit of apheresis platelets is transfused.

### Study endpoints

The primary efficacy endpoint is the proportion of patients transfused with any blood product (i.e., FFP, platelets or cryoprecipitate) prior to CVC. The primary safety endpoint is the incidence of major bleeding within the first 24 h after catheter insertion. Major bleeding was defined according to the HEME tool [41]. According to the HEME tool, major bleeding is defined as overt bleeding with any of the following (in the absence of other causes): decrease in hemoglobin of 20 g/L or more, transfusion of 2 or more units of RBC with no increase in hemoglobin level, decrease in systolic BP by 10 mmHg or more while the patient is sitting up, spontaneous decrease in systolic BP of 20 mmHg or more or increase in heart rate by 20 bpm or more; bleeding at any one of the following sites: intracranial, intraspinal, intraocular, pericardial, retroperitoneal or intraarticular; or wound-related bleeding requiring an intervention.

The secondary efficacy endpoints will be: the proportion of patients transfused with each blood component and the infused volume of each blood component, hemoglobin and hematocrit levels before and up to 24 h after CVC, costs associated to blood product transfusion and laboratory tests, ICU and hospital length of stay, and ICU, 28-day and hospital mortality. Secondary safety endpoints will include: the incidence of minor bleeding complications (i.e., oozing or hematoma with no criteria for major bleeding) and the proportion of patients transfused due to bleeding within the first 24 h after CVC, and the incidence of acute transfusion-related adverse events (i.e., transfusion-associated cardiac overload, acute hemolytic transfusion reactions, anaphylactic reactions, febrile nonhemolytic reactions and urticarial reactions). The transfusion of bleeding patients after CVC will follow the Hospital Israelita Albert Einstein institutional transfusion protocol. Accordingly, if INR >1.5 or aPTT >32 s, FFP (10 mL per kg) is administered. If serum fibrinogen <150 mg/dL, cryoprecipitate (1 unit per 10 kg) or fibrinogen concentrate (2 to 4 g) is administered. If the platelet count <50 x 10^3^/mm^3^, random platelets (1 unit per 10 kg) or apheresis platelets (1 unit) are administered. Correction of hypothermia (axillary temperature ≥35 °C), hypocalcemia (ionized calcium ≥1.14 mmol/l) and acidosis (pH ≥7.31) is always recommended.

### Participant timeline and study flowchart

**Fig. 4:**
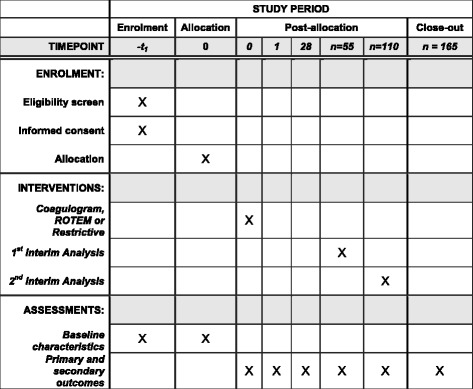
The Standard Protocol Items: Recommendations for Interventional Trials (SPIRIT) participant timeline in the POCKET trial. ROTEM®, rotational thromboelastometry

**Fig. 5:**
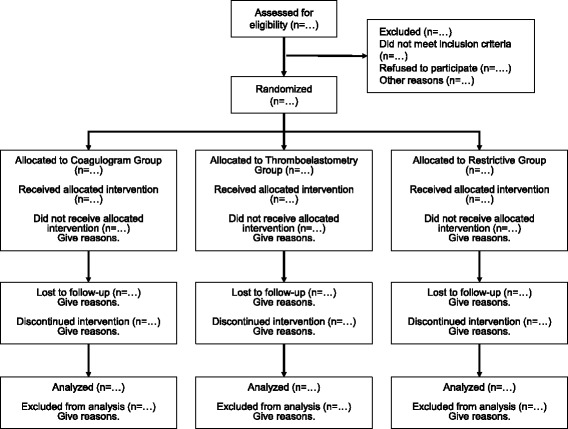
Study flowchart of the POCKET trial

The participant timeline and study flowchart are presented in Figs. 4 and 5, respectively. A filled Standard Protocol Items: Recommendations for Interventional Trials (SPIRIT) Checklist is available (see Additional file 1).

### Recruitment

Patients will be recruited in the two mixed medical-surgical ICUs of Hospital Israelita Albert Einstein in São Paulo, Brazil. On a daily basis, the research team will actively search the records and patient list for patients admitted with a diagnosis of cirrhosis. Additionally, on-call and horizontal care staff will be encouraged to recommend eligible patients to the research team at any time. The current recruitment rate is four patients per month with an expected duration of recruitment of 42 months. Recruitment rate will be monitored trimonthly.

### Randomization, allocation and blinding

Allocation sequence with random numbers will be computer-generated using random, varied size blocks and stratification by severe renal failure (i.e., hemodialysis program). The randomization sequence will be stored in individually separated and sequentially numbered opaque, sealed envelopes and kept secured in a locker with restricted access (centralized at ICU pharmacy). After the consent term is signed and coagulation tests collected, patients will be randomly assigned to study groups with an allocation ratio of 1:1:1.

Allocation concealment will be preserved by holding randomization sequence disclosure until the consent term is signed and coagulation tests collected. Patients will be enrolled by an on-call recruiter, and assignment to intervention will be provided by an independent researcher, who will be the only one to know which intervention the patient will be assigned to. The research subjects, central venous catheter inserter and outcome assessors will be blinded to the assigned intervention and bleeding will be assessed by the use of the HEME tool [41] immediately after CVC and 24 h after the procedure. The catheter inserter is not directly responsible for care of the patient included in the trial and will not have access to the patient’s chart. In case of major bleeding or serious transfusion-related adverse reaction, unblinding to the attending physician is anticipated.

### Sample size calculation

We calculated the sample size based on the primary efficacy endpoint. We assumed a transfusion rate of 90% in the Coagulogram-based group (i.e., control group) [11] and an absolute risk reduction of 25% for the Thromboelastometry-based and Restrictive groups. Using a study power of 0.80 and alpha of 0.05, we need *n* = 43 patients per group. The sample size was adjusted for multiple comparisons (additional increase of 20%, *n* = 8 per group) (Coagulogram-based versus Thromboelastometry-based, Coagulogram-based versus Restrictive, and Thromboelastometry-based versus Restrictive) according to Witte et al. [43]. To account for loss to follow-up, we have added 10% (*n* = 4) more patients to the adjusted sample size. The final required sample will be 55 patients per group (total estimated sample size of 165 patients).

### Data collection, monitoring and interim analysis

Trained researchers will be responsible for data collection. The principal investigator (PI) will be responsible for double-checking the collected data and input data into the study database. The quality of data will be guaranteed by locking the variables’ input range and random checking for quality 5% of patient data at each interim analysis and final analysis database.

The collected data will include age, race, gender, weight, hospital and ICU admission dates, main ICU diagnosis, cirrhosis etiology, transplant list status, comorbidities, Simplified Acute Physiology Score (SAPS 3) [44], Sequential Organ Failure Assessment (SOFA) score [45], Model for End-stage Liver Disease (MELD) score [46], transfusion of blood products in the previous 24 h and 24 h after CVC, procedure time, procedure indication, catheter type, number of attempts, insertion site, inserter, elective/emergency insertion, hemoglobin, hematocrit, PT, aPTT, platelet count, serum fibrinogen, ionized calcium, pH, peripheral temperature, ROTEM® parameters (CT, CFT, α angle, MCF and A10), type and quantity of transfused blood products for the procedure, adverse events related to the procedure and transfusion, when indicated, length of ICU and hospital stay and mortality at day 28.

An independent Data Safety Monitoring Board (DSMB) will conduct two interim analyses after the enrollment of 55 and 110 participants. The Lan-DeMets alpha spending function method will be used to determine statistical differences between groups [47]. After DSMB assessment, a report will be generated recommending whether the trial should continue or stop due to efficacy or harm. The final decision to stop the trial will be held by the PI together with the POCKET Trial Investigators. An independent audit will be conducted every 6 months after recruitment of the first patient. Every serious adverse event will be immediately reported to the Ethics Committee and the DSMB, and minor adverse events will be enlisted and periodically released. Every protocol amendment will be subject to approval from the local Ethics Committee.

### Statistical analysis plan

All analyses will be performed as intention-to-treat. Categorical variables will be summarized as absolute and relative frequencies. Normality will be tested with the Kolmogorov-Smirnov test. Accordingly, continuous variables with normal distribution will be summarized as mean ± SD, or median and interquartile range (IQR) in case of non-normal distribution.

Comparisons between groups of continuous variables will be performed with one-way analysis of variance (one-way ANOVA) followed by Tukey’s test for multiple comparisons (for normally distributed variables), or the Kruskal-Wallis test followed by the Mann-Whitney *U* test (for non-normally distributed variables). Proportions between groups will be analyzed with the chi-square test or Fisher’s exact test, as appropriate.

All binary outcomes will be analyzed using a generalized linear model with binomial distribution and logit link function followed by pairwise comparisons adjusted by Bonferroni correction for multiple comparisons. Pairwise comparisons will be presented as odds ratio (OR) along with 95% confidence interval (95% CI), which will be calculated by logistic regression.

A survival curve will be performed with the Kaplan-Meier estimator and the log-rank test will be used to compare groups. To assess time-to-event (28-day mortality), an unadjusted Cox proportional hazards regression model will be performed. The hazard ratio (HR) with its respective 95% CI will be reported.

Prespecified subgroup analysis for the primary outcome will be presented as a Forest plot, and will include the presence of hypothermia (<35 °C), SAPS 3 score (≥50 points) and MELD score (≥25 points). Interaction between groups and each covariate will be assessed by multivariate logistic regression.

Two-tailed tests will be used and when *p* < 0.05, the test will be considered statistically significant. To account for multiple comparisons (Coagulogram-based versus Thromboelastometry-based, Coagulogram-based versus Restrictive and Thromboelastometry-based versus Restrictive), the α level will be adjusted to 0.0167.

We will make maximum effort to reduce missing data. We will use the multiple imputation method to handle missing data when necessary. The Statistical Package for the Social Sciences version 22.0 (IBM Inc., Chicago, IL, USA) will be used for statistical analyses. GraphPad Prism version 6.07 (GraphPad Software Inc., La Jolla, CA, USA) will be used for graph plotting.

### Costs analysis

Transfusion and laboratory test costs will be assessed. For the analysis of the costs of laboratory tests, we will include the total cost of the test(s) (i.e., ROTEM® or SCTs), which will guide the blood transfusion. The unit cost information regarding blood products and coagulation tests will be obtained from the blood bank and main laboratory of Hospital Israelita Albert Einstein.

### Ethics

The study was designed in accordance to the Declaration of Helsinki. The Hospital Israelita Albert Einstein Ethics Committee approved the study on 12 August 2014 (reference number: 734824). Recruitment of participants started in September 2014 and is currently ongoing. The study was registered at ClinicalTrials.org (identifier: NCT02311985) on 3 December 2014. The research team will release the study results in scientific media regardless of their final results.

A trained research team member will obtain the informed consent term from the research subject or their next of kin. A deferred informed consent term will be applied in case of emergency CVC when the patient lacks clinical condition and no next of kin is immediately available. In this case, the definitive informed consent must be obtained within 24 h after the procedure. The participants may leave the study at any time with no treatment disadvantage. All study information will be kept secured with limited access. The patient database will be password-protected and patients will be identified by trial ID numbers. A translated copy of the Informed Consent Form is available (see Additional file 2).

## Discussion

Our trial is designed to compare three protocols to guide transfusion of blood components prior to CVC in critically ill patients with chronic liver failure. We hypothesized that restricted and thromboelastometry-guided transfusion protocols would be safe and significantly reduce empirical transfusion of blood products in cirrhotic critically ill patients. By reducing transfusion rates, these strategies would also reduce transfusion-related complications and costs. This would help to provide quality evidence for reduction of empirical transfusion of critically ill patients with coagulopathy in clinical practice.

One main focus of our trial is that the transfusion protocols used are based on a comprehensive assessment of coagulation, which includes the contribution of coagulation factors, platelets and fibrinogen. Most randomized trials published so far have relied on a specific aspect of coagulation and a specific blood product transfusion (e.g., FFP transfusion according to PT/INR) [48, 49]. Furthermore, fibrinogen is one of the most important coagulation factors [50], and we believe that including the evaluation of hypofibrinogenemia or dysfibrinogenemia would result in a more detailed understanding of coagulation status and, therefore, help in further prevention of bleeding. Additionally, we will use wide inclusion criteria, accounting for emergency insertions, active bleeding and use of antiplatelet aggregation agents making the case-mix of patients more similar to critical care practice.

We considered the use a restrictive blood transfusion protocol to be of vital importance. Several series of observational studies that included patients with coagulopathy have shown that experienced inserters have low complication rates in different populations of patients with coagulopathy, even when no blood transfusion is performed prior to the procedure [51–55]. A large meta-analysis showed that real-time ultrasound guidance for CVC significantly reduced failure to obtain central venous access, arterial puncture, hematoma formation and hemothorax by 82%, 75%, 70% and 90%, respectively, when compared to using anatomical landmark guidance [56]. Those positive effects are extended to hemodialysis catheters [57]. Furthermore, the use of real-time ultrasound guidance increases the success rate in the first attempt [6]. These may reduce complication rates to a minimum, even in patients with coagulopathy. We believe that blood product transfusion is frequently excessive when a trained inserter in ultrasound-guided CVC performs the procedure.

We have foreseen some potential issues for this trial. One major issue is not to complete the necessary recruitment required due to low adhesion by practicing physicians and the single-center setting. Every practice-changing protocol may be prone to resistance and low adhesion, mainly by cultural environment. Conversely, we will use broad inclusion criteria and health care provider sensitization campaigns to try to overcome this potential drawback. Another issue is an early trial stop due to either efficacy or harm. We programmed two interim analyses when one third and two thirds of patients are enrolled, respectively. Since few randomized clinical trials have assessed restrictive blood transfusion protocols prior to invasive procedures in critically ill patients with cirrhosis, we will follow the research subjects closely for adverse events.

This trial has potential limitations. Single-center trials may have a potential limited external validity [58] and may overestimate treatment effect when compared to multicenter trials [59]. The results from single-center trials must be applied with caution in daily clinical practice. This trial is not adequately powered to address transfusion-related complications, which ranges from one event in hundreds to thousands of blood transfusions [60]. Accordingly, our primary objective is to evaluate the feasibility and safety of strategies that use a more detailed overview of coagulation status or restrictive transfusion of blood components in critically ill patients with cirrhosis.

The results from this trial will help to develop multicenter randomized clinical trials to assess blood product transfusion prior to invasive procedures in critically ill patients with suspected coagulopathy. Our intention is to provide good-quality scientific evidence for transfusion of blood products in patients with coagulopathy, reducing excessive and empirical use of blood and costs in the health care system.

### Trial status

This trial is currently ongoing (recruitment phase).

## Additional files

Additional file 1: SPIRIT Checklist. (PDF 105 kb)
Additional file 2: Informed Consent Form. (PDF 102 kb)

## Abbreviations

95%CI: 95% Confidence interval
α: Alpha angle
A10: Amplitude at 10 min
aPTT: Activated partial thromboplastin time
CFT: Clotting formation time
CFT_EXTEM_: EXTEM’s clotting formation time
CT: Clotting time
CT_EXTEM_: EXTEM’s clotting time
CVC: Central venous catheterization
FFP: Fresh frozen plasma
HR: Hazard ratio
ICU: Intensive care unit
INR: International normalized ratio
IQR: Interquartile range
MCF: Maximum clot firmness
MCF_EXTEM_: EXTEM’s maximum clot firmness
OR: Odds ratio
PI: Principal investigator
PT: Prothrombin time
ROTEM®: Rotational thromboelastometry
SCTs: Standard coagulation tests
SD: Standard deviation
