# Supplementary material for: Thromboelastometry versus standard coagulation tests versus restrictive protocol to guide blood transfusion prior to central venous catheterization in cirrhosis: study protocol for a randomized controlled trial
Source: Trials. 2017 Feb 27;18:85. doi: 10.1186/s13063-017-1835-5 (PMC5327508; doi:10.1186/s13063-017-1835-5)
Supplement: Additional file 2: — Informed Consent Form. (PDF 102 kb) [file 13063_2017_1835_MOESM2_ESM.pdf]

HOSPITAL ISRAELITA ALBERT EINSTEIN

INFORMED CONSENT FORM

THROMBOELASTOMETRY VERSUS STANDARD COAGULATION TESTS VERSUS RESTRICTIVE PROTOCOL TO GUIDE BLOOD TRANSFUSION PRIOR TO CENTRAL VENOUS CATHETERIZATION IN CIRRHOSIS: A RANDOMIZED CLINICAL TRIAL

We are requesting from Mr./Ms. ____, or his/her legal representative, an authorization to participate in the study entitled: **Thromboelastometry versus standard coagulation tests versus restrictive protocol to guide blood transfusion prior to central venous catheterization in cirrhosis**. Participation in this study is completely voluntary. Your relative or the one you are representing (the patient) do not need to participate in this study by obligation. Consent to participate in this study may be withdrawn at any time by you or by the person you are representing without any disadvantage related to the treatment being provided by the hospital.

Why is this study being performed?

This study aims to understand the current practice of blood transfusion to correct coagulation alterations before insertion of catheter in a deep vein (e.g. the neck or the groin), which is also called central venous catheterization. These catheters are necessary to administer medications, i.e. antibiotics, pain medicine, medicine to control blood pressure, fluids etc. Additionally, it may be used to collect blood tests in patients with liver disease during admission to the Intensive Care Unit (ICU).

Patients with liver disease are believed to have coagulation disorders. This study is being performed because presently we don´t know which strategy is best to correct coagulation disorders before insertion of central venous catheters in patients with liver diseases.

The participant in this study will be submitted to insertion of central venous catheter in a deep vein. Additionally, according to the results of coagulation tests, blood transfusion may be necessary to correct alterations in coagulation before catheter insertion.

There are distinct strategies to correct the alterations in blood coagulation that will be used in this study, which include the following:

1. The standard coagulation tests – coagulogram. Coagulogram is currently the most utilized test to evaluate coagulation status.
    a. Use of coagulogram for blood transfusion according to a narrow limit
    b. Use of coagulogram for blood transfusion according to a broad limit
2. Rotational thromboelastometry. Thromboelastometry assesses coagulation in a faster and more comprehensive way when compared to the coagulogram.

With the results of this study, we aim to reduce blood transfusion before insertion of catheters in deep veins in patients with liver disease. Therefore, by reducing blood transfusion, we expect to reduce complications related to blood products, e.g. infection and transfusion reactions. Additionally, we will use ultrasound guidance to perform catheter insertion. Ultrasound guidance is safe and is associated with a significant decrease in complications related to catheter insertion.

What is the objective of this study?

The objective of this study is to compare three strategies to guide blood transfusion before insertion of catheters in deep veins in patients with liver disease admitted to the ICU. Actions taken to guide blood transfusion include: (1) thromboelastometry (new coagulation test), and coagulogram (standard coagulation test) with two different thresholds for transfusion: (2) current used limits and (3) broader limits.

What procedures will be performed in this study?

All participants in this study will be submitted to insertion of catheter in a deep vein. The participants may also receive blood components according to the strategy they will be allocated to. Currently, there is no best practice procedure to evaluate coagulation before insertion of catheters in deep veins. This study is being performed to answer this question.

Is catheter insertion part of this study?

No, it is not. Insertion of catheter will be indicated according to your physician recommendation. The catheter will be inserted according to the safety guidelines of Hospital Israelita Albert Einstein, which recommends ultrasound guidance to be used in every catheter insertion. What is being evaluated in this study is the best way to assess coagulation status and the need for blood transfusion to correct coagulation alterations before insertion of catheter in patients with liver disease. We are testing the best way to guide blood transfusion before catheter insertion.

What are the potential benefits of this study?

The major benefits related to this study are: reduction of blood transfusion and, consequently, its associated adverse reactions, i.e. allergic reactions, infection, fluid overload etc.

What are the potential risks of this study?

As describe before, this study intends to evaluate three strategies to guide blood transfusion before insertion of a central venous catheter. Therefore, the risks associated to participation in this study are related to bleeding. In case one blood transfusion protocol does not accurately reflect patient's need for transfusion, there may be less blood components transfused than the patient needs, resulting in a higher risk of bleeding. We reassure that insertion of a central venous catheter with ultrasound guidance carries a very low risk of bleeding (below 1%).

Is there any cost or reimbursement?

No, there is not. Participation in this study is not associated to any additional cost to you or the one you are representing, nor to the health insurance company. Therefore, there is no reimbursement or any kind of financial benefit.

In case I do not want to participate in the study, is there any other option?

You or the one you are representing may freely choose to consent, or not to participate in this study. If your option is not to participate, ICU care and all institutional routines will be followed without any disadvantage for the patient.

Besides, the attending physician may interrupt the study protocol at any time if he/she judges necessary. It is important to remember that insertion of the central venous catheter is indicated anyway. What will differ when participating in this study is the method used to assess coagulation alterations and the need for blood transfusion before catheter insertion. On the other hand, if you or the one you are representing do not wish to participate in this study, the standard way to assess coagulation alterations (coagulogram according to hospital protocol) will be used to guide blood transfusion.

Will my personal information and/or the results of my lab tests be kept confidential?

All data used for the purpose of this study will be kept in confidentiality. The results of this study will become available for academic or scientific purposes, without identifying any particular patient.

How long will I participate in this study?

In this study we will follow you up until hospital discharge, without performing any additional intervention. We will only collect data about your evolution in the ICU. During the period of this study, you may request us to clarify any doubts or give you any additional information. To do this, you may contact the study researchers (displayed below) or the Research Ethics Committee of Hospital Israelita Albert Einstein.

Can I withdrawal my participation in this study?

Participation in this study is absolutely voluntary. You may withdraw your consent to participate in this study at any time. This decision is not associated with any disadvantage related to care provided to you or the one you are representing.

You may spend the time necessary to read this consent term and decide if you or the one you are representing (the patient) want to participate in this study. Feel free to discuss with other relatives, friends or healthcare team.

The **Research Ethics Committee of Hospital Israelita Albert Einstein**, São Paulo – SP, approved this study protocol. If you have any doubts in regard to the rights of the clinical research participant or the involved ethical aspects of a clinical study, please feel free to contact the **Research Ethics Committee of Hospital Israelita Albert Einstein** through (11) 2151-3729 or **via email**: cep@einstein.br.

The research team is fully available to clarify any doubts that may occur before, during or after the study in the following phones:

- Leonardo Lima Rocha, MD, principal investigator: (11) 987087163
- Thiago Domingos Corrêa, MD PhD, co-investigator: (11) 996563506

I have read and understood the study objectives as well as every procedure that will be performed. I am aware of the possible risks and benefits. Should I have any additional doubts, I will contact the study research team.

Print Name of person granting the consent ________________________

Signature of person granting the consent ________________________

Date: _________________

Day/month/year

I confirm that the participant was given an opportunity to ask questions about the study, and all the questions asked by the participant have been answered correctly and to the best of my ability. I confirm that the individual has not been coerced into giving consent, and the consent has been given freely and voluntarily. A copy of this ICF has been provided to the participant.

Print Name of Researcher taking the consent ________________________

Signature of Researcher taking the consent ________________________

Date: _________________

Day/month/year
